# Paul Emmelkamp Becomes “Ambassador of Clinical Psychology and Psychological Treatment”

**DOI:** 10.32872/cpe.8303

**Published:** 2022-03-31

**Authors:** Maaike H. Nauta, Thomas Ehring

**Affiliations:** 1 Department of Clinical Psychology and Experimental Psychopathology, University of Groningen, Groningen, The Netherlands; 2 Department of Psychology, Clinical Psychology and Psychological Treatment, LMU Munich, Munich, Germany

Paul Emmelkamp is a scientist-practitioner *pur sang*. From the start of his career on, he has put an emphasis on the importance of integrating science and clinical practice, providing many important contributions to Clinical Psychology and Psychological Treatment in Europe and beyond.

**Figure:**
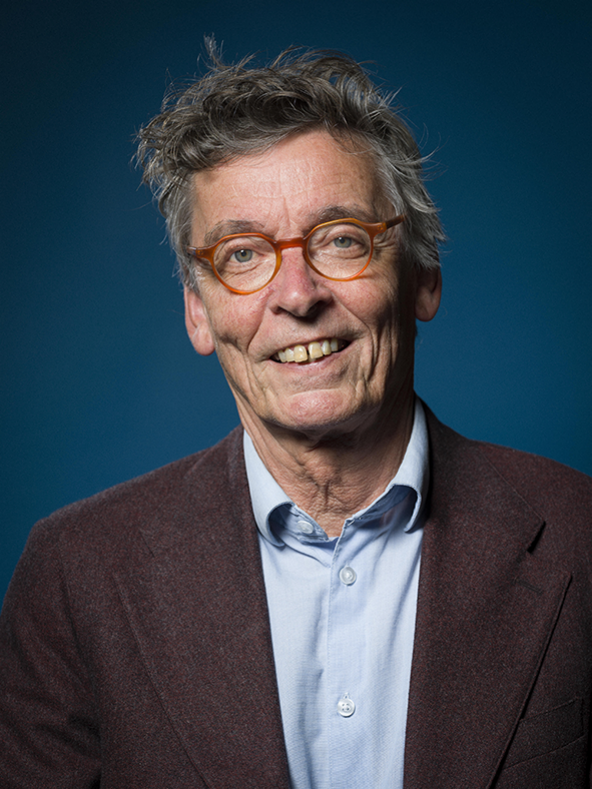
Paul Emmelkamp (2018) (Source: Paul Emmelkamp's own private collection)

In 1975, Paul obtained his PhD on ‘The behavioural treatment of agoraphobia’ from the University of Utrecht, where he had previously studied and completed his postdoctoral training in psychotherapy. He then moved to the University of Groningen, starting as an Assistant Professor and being appointed as a Full Professor in Clinical Psychology and Psychotherapy in 1986. Since 1996, he has been based as a University of Professor of Clinical Psychology at the University of Amsterdam. In 2006, Paul received the very prestigious appointment as Academy Professor of the Royal Academy of Arts and Sciences (KNAW). From 2013 to 2016, he then served as the Rector of the Netherlands Institute for Advanced Studies (NIAS). He is currently a fellow at the Institute for Advanced Studies in Paris.

The main focus of Paul’s research is to investigate the efficacy and effectiveness of psychological interventions, especially using randomized controlled trial methodology. Since the earlier 70s, he has published more 70 randomized controlled trials together with a large number of national and international collaborators. Since his early studies on agoraphobia, he has extended his work to cover other anxiety disorders, obsessive compulsive disorder, post-traumatic stress disorder, depression, burn-out, addiction, personality disorders, perpetrators of sexual violence, and childhood ADHD and behavioral problems. His work is not limited to studies on adult populations, but also includes studies on children, adolescents and the elderly. Having collaborated closely with him at different time points in his career (MN from 1996-2005; TE from 2007 – 2012), we would like to share some impressions of Paul Emmelkamp as a scientist-practitioner that we think make him an excellent ambassador for EACLIPT.

Readers may wonder what motivates a researcher to focus especially on one of the most challenging and time-consuming type of research in clinical psychology, i.e. mainly conducting clinical trials. In our impression, Paul’s motivation has always been to conduct research that matters, that has a real impact on clinical practice. In particular, he is driven to develop and test treatments that can work for many different patients, not just for the highly motivated “YAVIS” (young, attractive, verbal, intelligent, and successful) patients or students with elevated levels of psychopathology, but in particular for those who typically get referred to mental health institutions, often with a variety of comorbidity and a long duration of mental health problems. When embarking on his career, he perceived psychotherapy as too elitist, and it has always been his mission to have psychotherapy available for all in need, including “the man in the street”. Therefore, Paul has conducted many RCTs with “real-life” patients recruited within routine mental health settings, while at the same time ensuring rigorous methodology and the use of well-described treatment manuals.

To study the effectiveness of treatments, Paul made important contributions to manualizing treatments. He was the first in the Netherlands to break down treatments to manuals that were transparent and transferrable. The first aim was scientific: to define and consolidate the content of the treatments, so that therapists would adhere to the same set of interventions, and that patient would receive a similar treatment in one treatment condition in a trial. The side-effect of this has had a large impact on the field: once treatments were proven effective, they were transparently described, suitable for transfer to new therapists, and available for implementation. Paul has contributed to the dissemination of many of such manuals.

For Paul, the most important quest is to establish scientific evidence of the effectiveness of treatments, so that individuals with mental health problems can receive those treatments that have been proven effective. Even though he has mainly conducted studies on cognitive behavioral therapy, he is not necessarily identified with this specific treatment orientation. “I am fine with anything, as long as it works” (interview at the Dutch radio series Noorderlicht in 2003). He keeps looking for the evidence (and also for the non-evidence, as illustrated by the book “Failures in behavior therapy” co-edited with Edna Foa in 1983). As such, he likes to remain critical of the advances that have been made, to keep questioning things that seem “self-evident” without the data behind them, and to remain looking for further evidence. He is also not shy of – and even enjoys – raising controversial issues, playing the devil’s advocate, or pointing out that the emperor may actually not be clothed. If you are looking for a stimulating and controversial discussion about the state of clinical psychology, invite Paul to talk e.g. about the role of experimental psychopathology in clinical innovation, the use of non-clinical or analogue samples in clinical research, the ubiquitous claim of “novelty” in psychological interventions, or the rise of trademarked interventions. You may not necessarily agree with him on all these issues, but will certainly have a good and stimulating time!

On the other hand, when a new promising treatment or treatment format is developed, Paul may be among the first to start a trial investigating its efficacy. For example, he was one of the first to investigate e-health interventions and virtual reality therapy. He conducted trials investigating ACT or EMDR when many CBT-oriented researchers in Europe were still quite skeptical about these approaches. In addition, he has investigated interventions for mental health conditions that seem hard to implement, like interventions for sexual offenders in the context of a forensic clinic.

As a supervisor, Paul is and has been an inspiration to many, and has motivated many to continue in his tradition of studying treatment effectiveness. He has supervised 45 PhD students as well as numerous master students, bachelor students, and clinicians. He is a co-founder of the Research School "Experimental Psychopathology" (EPP), a graduate school and research network uniting EPP researchers from various universities in the Netherlands and Flanders, and was the Chair from its foundation in 1995 until 2014. The research school has contributed to the development of a strong and active research network. From the very beginning of his career, he has been building a strong network with colleagues across Europe and beyond, starting with a seminal collaboration with Edna Foa and Isaac Marks in the 70s. Similarly, he has also always been open to collaboration coming from outside of academia. When we were working with Paul in Groningen (MN) and Amsterdam (TE), most projects we collaborated on had actually been initiated by practitioners who wanted to answer a research question of relevance for their respective settings. Topics ranged from evaluating treatments for PTSD in adults or for oppositional behavior in children in routine clinical settings to studying psychological consequences of a severe earthquake on emergency personnel in Pakistan. His current fellowship in Paris focuses on mental health interventions for refugees, a very timely and societal relevant topic.

The networking and intensive collaboration that is characteristic of his research has certainly been facilitated by the fact that Paul is a very approachable, open, and warm person, with a brilliant sense of humor, and an open door. In addition, Paul has always been the opposite of a remote researcher in the ivory tower. Instead, he has continued seeing patients as a therapist throughout his career, has trained and supervised generations of students and therapists in conducting psychological treatment. He has also provided service to many different institutions, nationally as well as internationally, as a committee member, advisor, or board member, e.g., hosting the EABCT conference as president in the Netherlands (1987 and 2014), and serving as president of the president of the *International Federation for Psychotherapy* from 2014-2018. Last but not least, he is the founder of Clinical Psychology & Psychotherapy and has been its Editor since 1993.

We are confident Paul Emmelkamp will prove being a wonderful ambassador for EACLIPT.

